# Diurnal and Circadian Rhythms in the Tomato Transcriptome and Their Modulation by Cryptochrome Photoreceptors

**DOI:** 10.1371/journal.pone.0002798

**Published:** 2008-07-30

**Authors:** Paolo Facella, Loredana Lopez, Fabrizio Carbone, David W. Galbraith, Giovanni Giuliano, Gaetano Perrotta

**Affiliations:** 1 ENEA, Trisaia Research Center, Rotondella (MT), Italy; 2 ENEA, Casaccia Research Center, Rome, Italy; 3 BIO5 Institute for Collaborative Bioresearch and Department of Plant Sciences, University of Arizona, Tucson, Arizona, United States of America; Umeå Plant Science Centre, Sweden

## Abstract

**Background:**

Circadian clocks are internal molecular time-keeping mechanisms that provide living organisms with the ability to adjust their growth and physiology and to anticipate diurnal environmental changes. Circadian clocks, without exception, respond to light and, in plants, light is the most potent and best characterized entraining stimulus. The capacity of plants to respond to light is achieved through a number of photo-perceptive proteins including cryptochromes and phytochromes. There is considerable experimental evidence demonstrating the roles of photoreceptors in providing light input to the clock.

**Methodology:**

In order to identify genes regulated by diurnal and circadian rhythms, and to establish possible functional relations between photoreceptors and the circadian clock in tomato, we monitored the temporal transcription pattern in plants entrained to long-day conditions, either by large scale comparative profiling, or using a focused approach over a number of photosensory and clock-related genes by QRT-PCR. In parallel, focused transcription analyses were performed in *cry1a*- and in *CRY2-OX* tomato genotypes.

**Conclusions:**

We report a large series of transcript oscillations that shed light on the complex network of interactions among tomato photoreceptors and clock-related genes. Alteration of cryptochrome gene expression induced major changes in the rhythmic oscillations of several other gene transcripts. In particular, over-expression of *CRY2* had an impact not only on day/night fluctuations but also on rhythmicity under constant light conditions. Evidence was found for widespread diurnal oscillations of transcripts encoding specific enzyme classes (e.g. carotenoid biosynthesis enzymes) as well as for post-transcriptional diurnal and circadian regulation of the *CRY2* transcript.

## Introduction

Plants have adapted their growth and development to make use of the diurnal light/dark cycle. This is manifested at both the physiological level, with leaf and stomatal movements, modulation of growth, and photoperiodic flowering, and, at the molecular level, with diurnal cycles in enzyme and gene activity. The day/night cycling of gene expression is controlled, primarily, by light and temperature and, secondarily, by a free-running internal molecular timekeeper known as the circadian clock. The intimate connection between light signalling pathways and the circadian oscillator allows the anticipation of the environmental transitions and the measurement of day-length as an indicator of changing seasons.

Our current understanding of the plant circadian clock derives mostly from genetic studies in *Arabidopsis thaliana* and rice [1]. Commonly, the circadian clock system is divided into three parts [2]: an input pathway that entrains the clock, by transmitting light or temperature signals to the core oscillator, the central oscillator (the clock), responsible for driving 24-h rhythms, and the output signals that generate the fluctuation of a wide range of molecular, biochemical and developmental responses.

Plants have evolved several classes of photoreceptors to monitor their environmental light signals. These include red and far-red-light–absorbing phytochromes (PHYs) and UV-A/blue light–absorbing cryptochromes (CRYs) and phototropins [3], [4]. Recent evidence shows that UV-B and green light also affect plant development, but the sensing mechanisms underlying these responses have not been elucidated. Green light has been shown to affect plant processes via both cryptochrome-dependent and cryptochrome-independent pathways [5].

Genetic experiments with *Arabidopsis* mutants have established roles for PHYA, PHYB, PHYD, PHYE, CRY1 and CRY2 in the clock input pathway [6]–[8]. Light-labile PHYA is the predominant photoreceptor at low intensities of red and blue light, whereas PHYB and CRY1 predominate at high intensities of red and blue light, respectively [8]. Multiple photoreceptor mutants, such as *cry1 cry2* or *phyA phyB cry1 cry2* [6], retain rhythmicity and are still able to entrain the clock to a light-dark cycle, suggesting that photoreceptors can provide light input to the clock in a redundant fashion [9]. Also, novel putative photoreceptor families have been implicated in circadian responses, such as the ZTL/FKF/ LKP2 family [10]–[13].

Considerable evidence exists for interaction among photoreceptors. For instance, PHYA and CRY1 directly interact at the molecular level, with CRY1 serving as a phosphorylation substrate for PHYA *in* vitro [14]. *In vivo*, CRY1 is phosphorylated in response to red light in a far-red reversible manner [14]. A *cry1* null mutant shows lengthened period in low intensity red or white light, and there is no additivity seen in the double *phyA cry1* mutant [6]. This suggests that CRY1 acts as a signal transduction component downstream from PHYA in the low intensity light input pathway to the clock [15].

Genetic studies have implicated two other genes, *EARLY FLOWERING 3* (*ELF3*) and *GIGANTEA* (*GI*), in light signalling to the clock. *elf3* loss-of-function alleles result in early flowering, hypocotyl elongation, and conditional arrhythmicity in continuous light [16], [17]. Genetic experiments suggest substantial redundancy in ELF3 and PHYB function [18]. ELF3 interacts with PHYB and seems to act as a negative modulator of PHYB signalling to the clock, as *ELF3* overexpression both lengthens the circadian period and attenuates the resetting effects of red light pulses, whereas loss of *ELF3* function renders the plant hypersensitive to light signals [16], [17], [19].

In *Arabidopsis*, *GI* positively regulates expression of the flowering time genes *CONSTANS* (*CO*) and *FLOWERING LOCUS T* (*FT*). *GI* encodes a nucleoplasmically localized protein that mediates a number of responses, such as photoperiodic flowering, circadian rhythms and phytochrome/cryptochrome signalling [20]. The key roles played by *GI* are evident when analyzing the effect of *gi* mutants over leaf movement and gene expression rhythms of multiple clock controlled and flowering genes, including *GI* itself [21], [22].

In-depth studies on *Arabidopsis* have begun to shed light on the molecular mechanisms underlying the functioning of the circadian clock. The current best candidates for *Arabidopsis* clock components are CIRCADIAN CLOCK ASSOCIATED 1 (CCA1) and its redundant homolog LATE ELONGATED HYPOCOTYL (LHY), which are transcription factors containing a single MYB domain [23]–[25]. Furthermore, pseudo-response regulators (*PRR*), *GI*, *ZEITLUPE/ADAGIO* (*ZTL/ADO1*), *LOV KELCH PROTEIN* (*LKP2*), *EARLY FLOWERING 3* and *4* (*ELF3* and *ELF4*), *LUX ARRHYTHMO/PHYTOCLOCK* (*LUX/PCL1*), *TIME FOR COFFEE* (*TIC*), *SENSITIVITY TO RED LIGHT REDUCED* (*SRR1*) and *TEJ* [26], [27], have also been involved in the circadian machinery.

The clock mechanism in *Arabidopsis* was first proposed to comprise a feedback loop, in which two partially redundant genes, *LHY* and *CCA1*, repress the expression of their activator, TOC1 [28]. This circuit cannot fit all experimental data [29], as a short-period rhythm persists for several cycles both in *lhy cca1* [30], [31] and in *toc1* mutant plants [32]. Subsequently, many other clock-associated genes have been identified and incorporated into the simple model, resulting in a somewhat complicated interlocking multiloop model, comprising the feedback loop between LHY, CCA1, and TOC1, and a predicted, interlocking feedback loop involving TOC1 and a hypothetical component Y [31]. The model was recently extended, suggesting GI as a candidate for Y and including a feedback loop between PRR7, PRR9 and LHY\CCA1, giving rise to a three loop circuit [33]. Analysis of the three-loop network suggests that the plant clock consists of morning and evening oscillators, coupled intracellularly, which may be analogous to coupled, morning and evening clock cells in *Drosophila* and mouse [33].

Light signals typically trigger rapid changes in the mRNA levels of transcription factors, but the position that they occupy in a putative transcriptional cascade, and the steps interposed between the photoreceptors and the first row of transcription factors, have not been fully established [34]. Recent work using an expressed sequence tag (EST)-based DNA microarray has suggested that nearly one-third of the genome is regulated in white light. In addition, the genome expression patterns largely overlap in 6-day-old seedlings grown under white, far-red, red, and blue light. More than 26 cellular pathways, ranging from DNA replication to transcription, metabolism, protein degradation, plant defence, and developmental regulation, have been found to be redundantly regulated by all light signals [35].

Furthermore, Schaffer and collaborators observed that 11% of genes showed differential expression at one or more of the phases tested during a light/dark cycle [36]. A large fraction of *Arabidopsis* genes that showed diurnal regulation was also circadian-regulated, as revealed by differential transcript abundance under constant light conditions [37], [36]. Oligo-based microarray experiments on *Arabidopsis* [37] allowed the detection of circadian oscillations in mRNA abundance of 5–6% of the 8200 genes examined. In tomato, several photosynthesis-associated genes, including *RBCS*, *LHCI* and *LHCII*, *PSAD*, and *OEE1*, were shown to be regulated in a circadian fashion through Northern blot and nuclear run-on experiments [38].

Here, we report the characterization of temporal transcript oscillations within the tomato genome using the novel, long oligo-based TOM2 microarray. Focused Real Time RT-PCR analyses over photoreceptor gene transcripts in both wild type tomato and genotypes with altered cryptochrome gene expression provided useful information about possible functional interactions between cryptochromes and the circadian clock machinery, as well as on regulatory interactions between different photoreceptors.

## Results

In order to identify transcripts showing temporal rhythmicity and to establish possible functional relations between photoreceptors and the circadian clock machinery in tomato, we performed extensive transcription analyses using the TOM2 microarray and using Quantitative Real-Time PCR (QRT-PCR) of the *PHYA*, *PHYB1*, *PHYB2*, *PHYE*, *PHYF* [39] and *CRY1a*, *CRY1b*, *CRY2* genes [40], [41]. Additional genes, already known to be regulated by the circadian clock in other plant species, including *GI* and *LHC*, were also investigated by QRT-PCR [21], [38].

Wild-type (wt) tomato plants were grown under a light cycle of 16h light/8h darkness (LD), as described in Materials and Methods, and sampled every 4h for 24 hours. Because diurnal changes of gene expression frequently reflect an underlying circadian rhythm, tomato plants entrained in LD were transferred to light constant conditions (LL), and then gene expression was monitored for additional 40 h at 4h intervals. Two genotypes with altered cryptochrome gene expression, *cry1a*- and *CRY2-OX* [42], [43], were also included in the experimental scheme.

To classify the time points at which the sampling was carried out, we used Zeitgeber time (ZT), that is defined as the time in hours from the start of a normal 16 h light–8 h dark cycle [44].

### Transcriptional profiling using the TOM2 microarray

We hybridised the TOM2 microarray with target RNAs extracted from ZT0 (presumptive dawn), ZT8 (eight hours after dawn), ZT16 (presumptive dusk) and ZT20 (four hours after dusk), in LD conditions (see Materials and Methods). The experimental design compared three time points to ZT0 used as a common reference: ZT8 vs. ZT0, ZT16 vs. ZT0 and ZT20 vs. ZT0.

**Figure 1:**
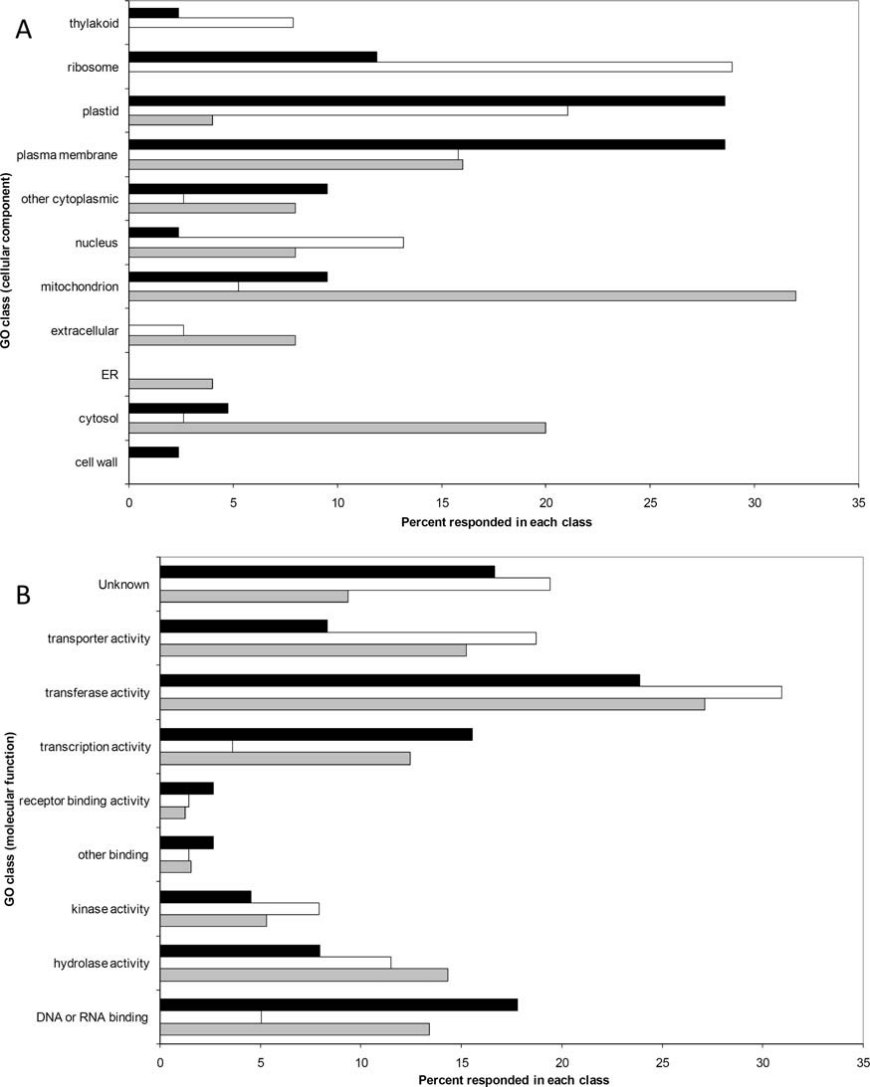
The impact of diurnal transcript oscillations on different categories of genes based on Gene Ontology. The percentages of diurnally regulated genes classified by cellular compartment (A) and by molecular function (B). Black bars: ZT20/ZT0. White bars: ZT16/ZT0. Grey bars: ZT8/ZT0.

Transcripts corresponding to microarray spots which passed ANOVA test at ZT8/ZT0, ZT16/ZT0, ZT20/ZT0 and showing an expression difference greater than three-fold in at least one of the time points (see Materials and Methods) were classified as diurnally regulated. According to this criterion, 1016 transcripts showed a diurnally regulated expression pattern, corresponding to 15% of all spots (6953) which passed quality controls (see Materials and Methods). Compared to their expression at dawn (ZT0), the majority of the genes coding for mitochondrial and cytosolic proteins were up-regulated in the middle of the light phase (ZT8) while the genes coding for ribosomal, nuclear and thylakoid proteins were preferentially more expressed at dusk (ZT16) (Figure 1A). Many genes coding for cytoplasmic membrane, cell wall and plastid proteins showed an up-regulation in the dark phase (ZT20) (Figure 1A). Regarding their molecular function, several genes coding for proteins involved in “transporter and transferase activity” were preferentially more expressed at dusk (ZT16), while two thirds of the genes up-regulated at ZT20 are associated with transcriptional control (“transcription factor activity and DNA or RNA binding” categories) (Figure 1B).

**Figure 2:**
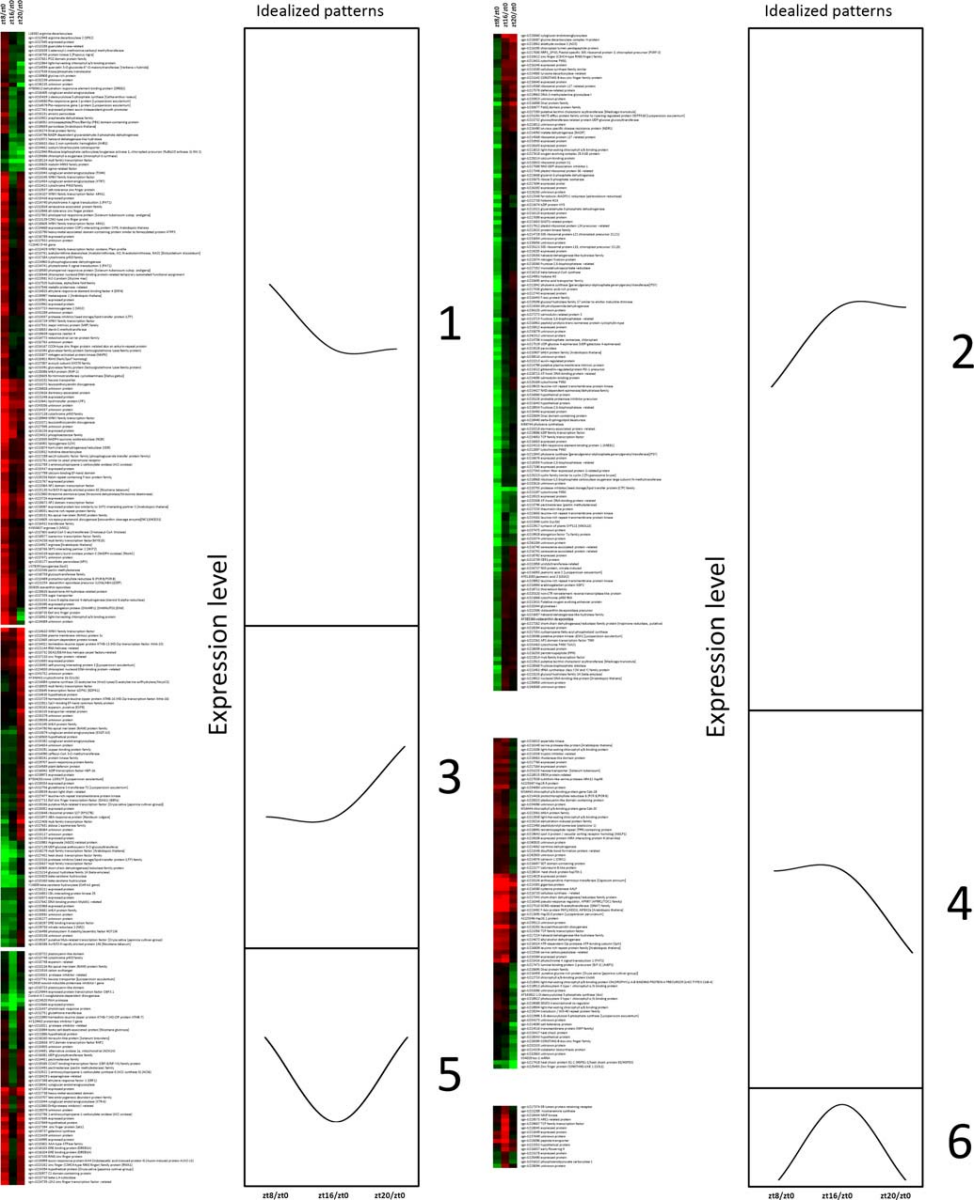
Clustering analysis of diurnally regulated genes (P ≤ 0.05). Idealized graphs, representing patterns of expression at ZT8/ZT0, ZT16/ZT0 and ZT20/ZT0, are shown.

In order to identify representative expression patterns of day/night regulated genes, we performed cluster analysis according to similarity of gene expression patterns at ZT8/ZT0, ZT16/ZT0 and ZT20/ZT0. Among transcripts with higher relative accumulation in the middle of the light phase (ZT8) (Figure 2, cluster 1) or both at ZT8 and at the presumptive dusk (ZT16) (Figure 2, cluster 4), we found many stress-responsive genes, such as genes coding for peroxidases, caspases, salt tolerance proteins, oxygenases and some members of the WRKY family [45]. Other transcripts in these clusters are involved in circadian rhythms, light signal transduction and flowering - *PSEUDO-RESPONSE REGULATOR 7* (*PRR7*), *FLAVIN-BINDING*, *KELCH REPEAT*, *F-BOX 1* (*FKF1*), *CONSTANS-LIKE 1* (*COL1*), and the flowering time gene *GI* [46], [11], [21]. Finally, these clusters included a number of genes implicated in the light-harvesting reactions of photosynthesis that, as expected, are more expressed in the light phase.

The converse clusters (Figure 2, cluster 3 and cluster 2) represent transcripts relatively more abundant in the dark phase (ZT20) or up-regulated at dusk (ZT16) and persisting at ZT20. These clusters contain elements involved in cellular communication and signal transduction and several transcripts involved in glycolysis/glycogenolysis and in the tricarboxylic acid cycle. We have also found some genes involved in the carotenoid pathway and several transcription factors of the WRKY, MYB, bHLH, leucine zipper, and zinc finger families.

Transcripts with a peak or a trough at the presumptive dusk (ZT16) are grouped, respectively, in cluster 6 and cluster 5 (Figure 2). Like for cluster 1, these clusters contain several genes coding for stress responsive-proteins. In particular protease inhibitors, endotransglycosylases, the cold stress responsive protein DREB1A [47] and a cell death-associated protein decreased at presumptive dusk (ZT16), while transcripts peaking at dusk comprise the circadian clock related gene, *ELF4* [48].

Several transcripts are differentially regulated at least three-fold simultaneously at all time points (ZT8, ZT16 and ZT20) (Student's t test P≤0.05) with respect to presumptive dawn (ZT0) (Table 1). Among the 27 transcripts with higher expression at ZT0 we found elements related to light signalling and flowering (Table 1B). These include a MYB-related transcription factor, *LATE ELONGATED HYPOCOTYL* (*LHY*), a member of the YABBY family (*ABNORMAL FLORAL ORGANS* (*AFO*)), *CONSTANS-LIKE* 1 (*COL1*), *SUPPRESSOR OF PHYTOCHROME A* (*SPA1*), and *EARLY LIGHT INDUCIBLE* (*ELIP*) genes [49]–[52]. Several of the 37 transcripts with lower expression at ZT0 are related to stress responses and hormone pathways (Table 1A). As expected, the *PSEUDO-RESPONSE REGULATOR* 7 (*PRR7*) transcript is also less expressed at dawn [46].

**Table 1:** Transcripts up-regulated (A) and down-regulated (B) at ZT0

zt8/zt0		zt16/zt0		zt20/zt0			A
Ratio	P-value	Ratio	P-value	Ratio	P-value	Gene_ID	Annotation
40,676	1,95E-02	37,195	4,81E-02	3,877	1,54E-02	sgn-U216348	APRR7 (APRR1/TOC1 family)
24,081	4,26E-03	46,603	1,13E-03	6,864	2,26E-03	sgn-U227341	short-chain dehydrogenase/reductase family protein
23,893	2,95E-03	5,277	2,44E-05	5,734	8,85E-04	sgn-U243036	unknown protein
21,624	1,73E-02	11,585	1,59E-02	3,264	1,18E-03	sgn-U214829	expressed protein
17,494	5,50E-04	3,399	3,04E-05	3,174	4,03E-03	sgn-U221348	expressed protein
17,069	1,99E-02	6,311	1,08E-02	3,050	2,09E-02	sgn-U231435	O-methyltransferase family 2 [Arabidopsis thaliana]
16,724	1,77E-02	8,194	8,13E-03	3,486	6,39E-03	sgn-U213589	protease inhibitor/seed storage/lipid transfer protein (LTP) family
15,621	3,66E-02	9,834	1,67E-02	8,262	3,62E-03	sgn-U214471	hydrolase, alpha/beta fold family
15,516	8,20E-03	10,086	2,04E-02	5,105	2,61E-02	sgn-U214470	hydrolase, alpha/beta fold family
15,269	1,02E-03	10,456	1,82E-03	5,413	6,03E-03	sgn-U218302	wound-responsive protein -related
12,718	1,21E-03	15,622	1,29E-02	6,601	2,23E-02	sgn-U233539	unknown protein
12,709	2,02E-02	12,884	2,01E-03	3,550	1,60E-03	sgn-U216720	cellulose synthase catalytic subunit
11,701	1,19E-02	3,192	3,78E-02	5,116	2,21E-02	sgn-U215735	heavy-metal-associated domain-containing protein
10,576	2,96E-02	10,126	1,65E-02	7,662	1,66E-03	sgn-U226639	cysteine protease XBCP3
10,292	2,30E-03	3,273	2,51E-02	9,745	1,96E-02	sgn-U222678	ABC transporter family protein similar to ABC1 protein
10,171	3,45E-02	3,181	8,48E-03	3,391	2,79E-02	sgn-U220461	unknown protein
9,650	1,78E-02	3,970	3,15E-03	6,221	2,55E-06	sgn-U213637	WRKY family transcription factor DNA-binding protein 4 WRKY4
9,611	1,51E-03	4,667	2,63E-02	3,541	1,27E-02	sgn-U217986	patatin-like latex allergen [Hevea brasiliensis]
9,092	1,10E-03	11,458	3,00E-04	3,311	6,37E-03	sgn-U220022	CLH2_ARATH Chlorophyllase 2
8,844	1,46E-02	6,183	9,89E-03	3,503	8,60E-03	sgn-U213926	drought-induced protein SDi-6 -
8,427	1,26E-02	6,856	6,09E-04	5,731	3,95E-03	sgn-U212562	glutamate decarboxylase 1 (GAD 1)
8,292	3,03E-02	4,683	1,71E-02	3,999	1,13E-02	sgn-U225595	AUX/IAA family
7,781	3,55E-03	3,635	2,40E-03	3,167	1,91E-04	sgn-U213578	BEL1-related homeotic protein 11 [Solanum tuberosum]
7,479	1,53E-02	6,450	2,52E-02	8,916	4,75E-03	sgn-U222728	senescence-associated protein -related
7,392	1,01E-03	3,554	6,16E-03	3,866	6,33E-03	sgn-U216076	receptor serine/threonine kinase PR5K [Arabidopsis thaliana]
7,323	1,37E-02	3,318	2,05E-02	6,838	9,15E-05	sgn-U224875	heat shock protein family
6,728	2,91E-02	5,074	6,38E-03	3,774	2,95E-03	sgn-U216827	cysteine proteinase
6,554	2,01E-03	8,854	3,68E-03	3,714	1,33E-02	sgn-U216414	unknown protein
5,751	4,52E-02	3,631	7,63E-03	3,077	1,24E-03	sgn-U213519	dehydration-induced protein (ERD15)
5,290	4,44E-03	7,321	2,52E-03	3,511	2,60E-04	sgn-U218536	serine/threonine protein kinase
5,208	4,04E-02	3,078	6,31E-03	3,248	2,52E-02	sgn-U232570	unknown protein
5,179	6,48E-03	3,203	4,97E-03	4,092	4,23E-03	sgn-U212706	light regulated protein -related
4,860	8,15E-03	4,314	1,92E-02	4,049	1,99E-03	sgn-U219226	senescence-associated protein
3,926	3,33E-02	4,668	3,77E-03	3,059	9,41E-03	sgn-U229252	nitrate transporter NRT1-1 [Glycine max]
3,864	1,81E-02	3,788	2,82E-02	3,152	2,06E-02	sgn-U214216	putative steroid membrane binding protein [Oryza sativa (japonica cultivar-group)]
3,697	9,00E-03	5,706	1,16E-02	10,814	9,84E-04	sgn-U215359	1-aminocyclopropane-1-carboxylate oxidase homolog (Protein E8)
3,071	1,25E-02	3,300	2,47E-02	8,554	7,00E-03	sgn-U217504	zinc finger (C3HC4-type RING finger) family protein (RMA1)

zt8/zt0		zt16/zt0		zt20/zt0			B
Ratio	P-value	zt16	P-value	zt20	P-value	Gene_ID	Annotation
0,004	1,16E-04	0,046	8,98E-05	0,193	5,89E-04	sgn-U218628	protein kinase family
0,001	7,75E-05	0,072	2,18E-03	0,251	4,60E-06	sgn-U227452	heat stress transcription factor HSFA9 [Helianthus annuus]
0,009	7,76E-05	0,119	1,17E-04	0,155	2,10E-04	sgn-U220755	protease inhibitor/seed storage/lipid transfer protein (LTP) family
0,026	3,27E-07	0,009	8,04E-09	0,021	2,25E-08	BT012912	Lycopersicon esculentum clone 114030R
0,044	4,58E-04	0,094	1,45E-02	0,018	1,52E-05	sgn-U237511	LHY
0,048	2,51E-05	0,067	2,03E-06	0,181	1,05E-05	sgn-U227174	pectinesterase family
0,054	9,85E-07	0,142	8,72E-06	0,299	1,38E-04	sgn-U226241	L-aspartate oxidase family
0,082	1,55E-07	0,099	3,47E-06	0,010	9,01E-08	sgn-U217418	heat shock protein 81-1 (HSP81-1/heat shock protein 83/HSP83)
0,085	2,63E-06	0,005	2,64E-06	0,248	1,85E-04	sgn-U213666	expressed protein
0,085	3,42E-06	0,272	1,45E-03	0,152	2,15E-05	sgn-U218682	abnormal floral organs protein (AFO) (FIL) YABBY1 (YABBY1)
0,087	2,09E-06	0,087	4,49E-06	0,286	3,94E-04	sgn-U215829	beta-carotene hydroxylase
0,090	2,96E-06	0,177	7,57E-05	0,109	5,24E-06	sgn-U222349	expressed protein
0,097	1,03E-06	0,163	1,16E-05	0,256	3,41E-05	sgn-U222883	phytochrome A supressor spa1
0,104	4,28E-05	0,177	4,42E-05	0,083	1,70E-05	sgn-U216986	DnaJ protein AtJ3
0,130	1,02E-04	0,035	1,74E-05	0,071	1,76E-07	sgn-U212621	AMP-dependent synthetase and ligase family
0,140	1,13E-04	0,041	8,67E-03	0,169	6,02E-06	sgn-U214765	copper-binding protein family
0,188	2,52E-06	0,128	6,98E-06	0,132	1,31E-05	sgn-U222990	expressed protein
0,198	2,22E-04	0,182	2,45E-04	0,278	8,19E-04	sgn-U217455	linker histone protein -related
0,215	3,86E-04	0,128	1,22E-06	0,144	2,38E-05	AY547273	early light inducible protein (ELIP)
0,216	8,70E-06	0,223	7,48E-05	0,285	2,17E-06	sgn-U222868	phytochelatin synthetase
0,218	1,29E-05	0,029	1,13E-02	0,091	4,92E-05	sgn-U224520	FtsH protease
0,240	1,21E-04	0,111	3,91E-02	0,244	2,01E-04	Control-4	2-oxoglutarate-dependent dioxygenase
0,249	2,15E-05	0,325	1,00E-04	0,323	6,23E-05	sgn-U215717	omega-6 fatty acid desaturase
0,267	2,02E-03	0,060	1,72E-03	0,021	6,00E-05	sgn-U218114	myb family transcription factor [Arabidopsis thaliana]
0,270	2,49E-04	0,130	5,06E-06	0,132	1,18E-05	sgn-U221454	ammonium transporter
0,320	2,19E-06	0,145	1,02E-06	0,097	2,92E-06	sgn-U217678	delta 9 acyl-lipid desaturase (ADS1)
0,324	1,92E-03	0,137	2,81E-03	0,006	3,73E-07	sgn-U225455	CONSTANS-LIKE 1 (COL1)

**Figure 3:**
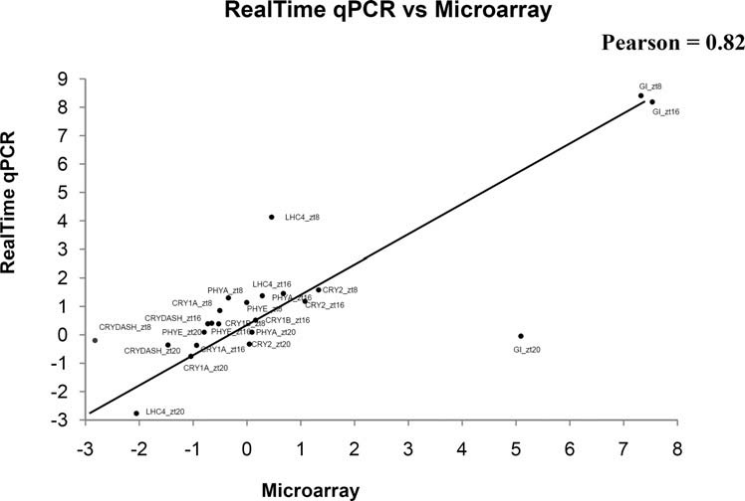
Pearson's correlations between gene expression levels of photoreceptors determined by QRT-PCR and the TOM2 microarray.

Transcription of cryptochrome and phytochrome photoreceptor genes was detected on the microarray. These genes were further assayed in depth by QRT-PCR with similar results (Figure 3). Given the higher resolution and sensitivity of the latter assay, we report the QRT-PCR data below.

### Diurnal mRNA oscillations of tomato photoreceptor genes

**Figure 4:**
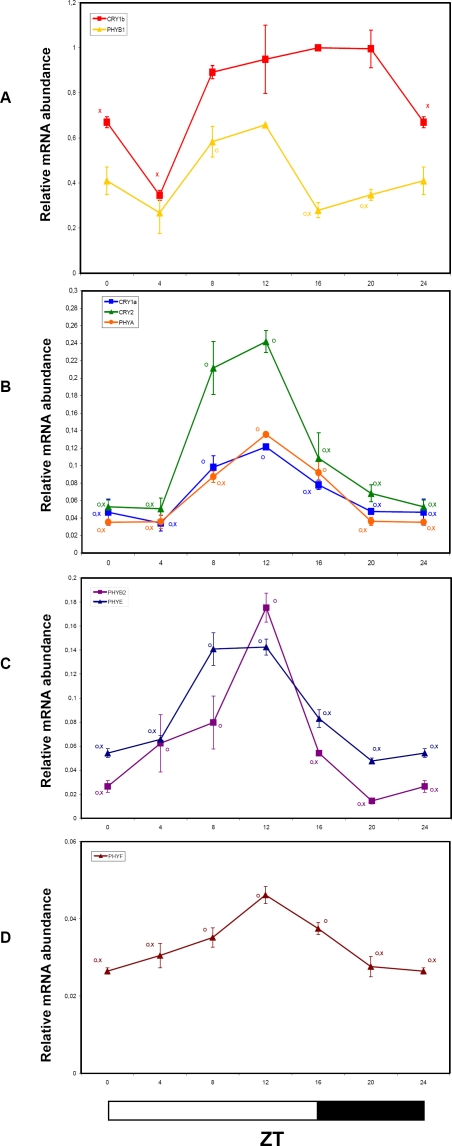
Diurnal oscillations of Cryptochrome and Phytochrome transcripts analyzed by QRT-PCR in tomato plants grown in LD conditions. Results are presented as a proportion of the highest value after normalization with β-actin. Open and closed bars along the horizontal axis represent light and dark periods, respectively. Time points are measured in hours from dawn (zeitgeber Time [ZT]). Data shown are the average of two biological replicates, with error bars representing SEM. Time points of *CRY1a*, *CRY2*, *PHYA*, *PHYB1*, *PHYB2*, *PHYE* and *PHYF* transcripts significantly different from the corresponding ones of the *CRY1b* gene (Student's t test, P ≤ 0.05) are marked with an O. Time points significantly different from the highest transcription value (Student's t test, P ≤ 0.05) are marked with an X.

We measured, by QRT-PCR, changes of mRNA accumulation of tomato phytochrome (*PHYA*, *PHYB1*, *PHYB2*, *PHYE*, *PHYF*) and cryptochrome (*CRY1a*, *CRY1b*, *CRY2*) transcripts under LD at 4-h intervals for 24 h. Although with differences in amplitude, most of the tomato photoreceptor transcripts showed diurnal fluctuations, reaching maximum expression between ZT8 and ZT12, and, with the exception of *PHYB1*, declined significantly during the dark period (Figure 4). The absolute expression and amplitude of the fluctuations of *PHYF* were significantly reduced relative to the other tomato photoreceptors (Figure 4D). The expression pattern of *CRY1b* was somewhat divergent from the other photoreceptors (Figure 4A). Only in early-morning (ZT4), *CRY1b* transcripts showed a trough (Figure 4A). *CRY1b* was also the most abundant photoreceptor transcript among those tested, remaining high throughout the 24h period.

In general, comparative analyses of diurnal expression pattern of phytochrome and cryptochrome genes showed qualitatively comparable oscillation phases, though significant differences in mRNA abundance were detected throughout the 24h period (Figure 4). Concerning the overall amplitude of oscillations, photoreceptor transcripts showed modest fold-changes, ranging from about 2× (*PHYF*) to 7× (*PHYB2*) (Figure 4), compared to other diurnally regulated and circadian genes like *LHC4* and *GI* (see below).

### Circadian rhythmicity of tomato photoreceptor transcripts in LL conditions

One of the diagnostic features of circadian rhythms is that they persist under constant light or darkness conditions. To determine whether the rhythmic fluctuations of the photoreceptor transcripts observed in LD conditions were maintained also in LL conditions, we measured their transcription in plants transferred to LL, after entraining the clock in LD. Samples were harvested at 4h intervals during a period of 40 h.

**Figure 5:**
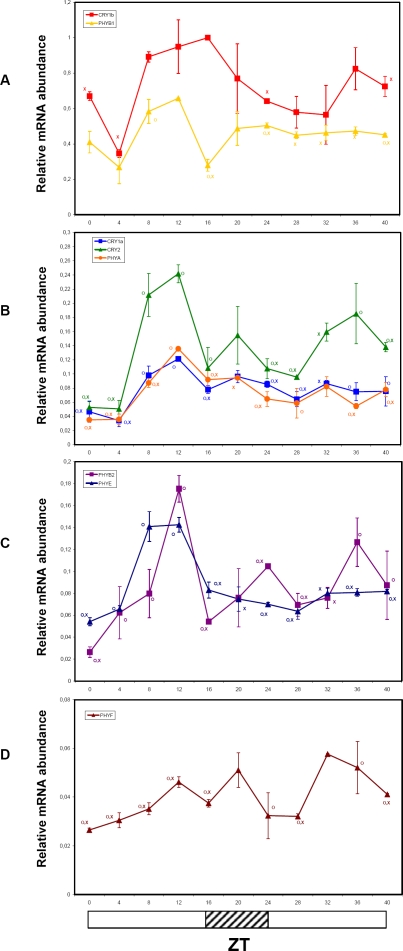
Circadian oscillations of Cryptochrome and Phytochrome transcripts in tomato plants entrained in LD conditions and then transferred to LL. Results are presented as a proportion of the highest value after normalization with β-actin. Open and hatched bars along the horizontal axis represent light and subjective night periods, respectively. Time points are measured in hours from dawn (zeitgeber Time [ZT]). Data shown are the average of two biological replicates, with error bars representing SEM. Time points of *CRY1a*, *CRY2*, *PHYA*, *PHYB1*, *PHYB2*, *PHYE* and *PHYF* transcripts significantly different from the corresponding ones of the *CRY1b* gene (Student's t test, P ≤ 0.05) are marked with an O. Time points significantly different from the highest transcription value (Student's t test, P ≤ 0.05) are marked with an X.

Under LL, transcript levels of *CRY1b*, *CRY2*, and *PHYB2* continued to cycle, suggesting that the circadian clock controls the expression of these genes (Figure 5). *PHYB1* and *PHYE* transcripts lost any detectable oscillation, while *PHYF* showed increased oscillation, compared to LD conditions.

### Effects of cryptochrome gene perturbation on expression of tomato photoreceptor genes in LD and LL

**Figure 6:**
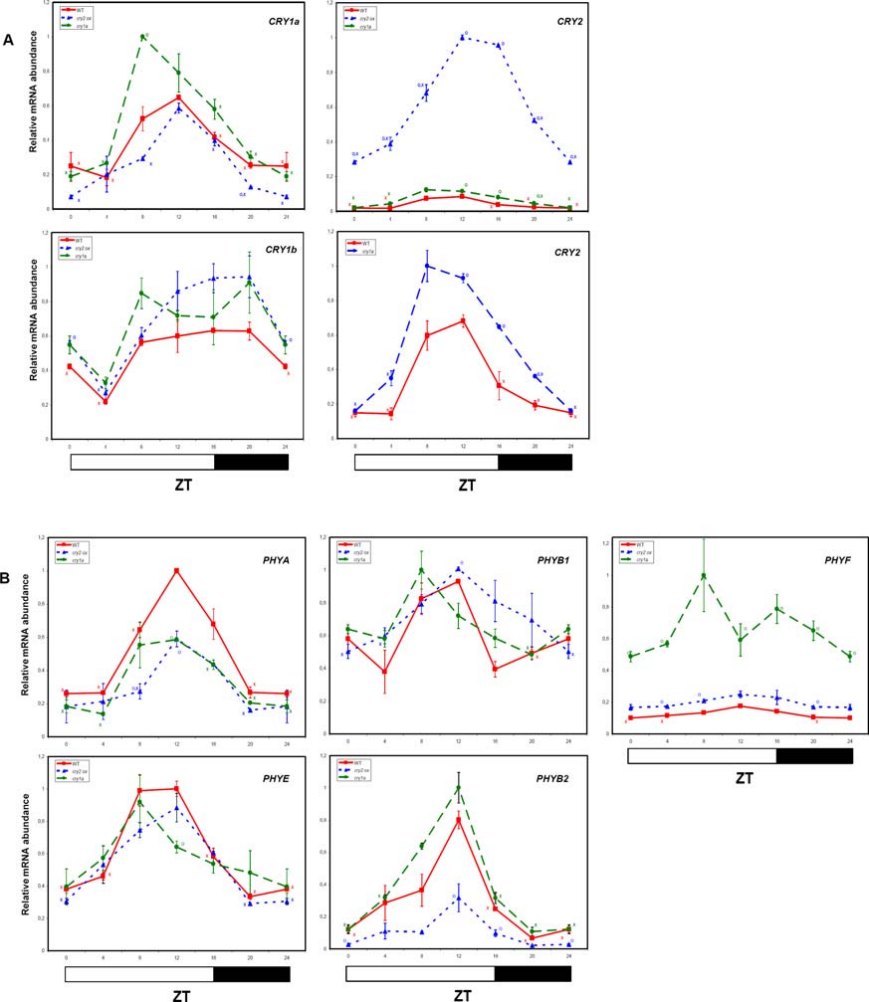
Effect of *CRY1a* loss-of-function and *CRY2* over-expression on diurnal expression of tomato cryptochrome (A) and phytochrome (B) genes. Wt, *cry1a*- and *CRY2-OX* tomato plants were grown under LD conditions. The abundance of the mRNAs were measured by QRT-PCR. Results are presented as a proportion of the highest value after normalization with β-actin. Open and closed bars along the horizontal axis represent light and dark periods, respectively. Time points are measured in hours from dawn (zeitgeber Time [ZT]). An additional panel depicts *CRY2* transcript values in wt and *cry1a*- genotypes to avoid the masking effect of *CRY2-OX* values. Data shown are the average of two biological replicates, with error bars representing SEM. Circles (O) indicate time points of *CRY2-OX* and *cry1a-* genotypes, significantly different from the corresponding ones in wt genotype (Student's t test, P ≤ 0.05). For each genotype X indicate time points significantly different from the highest transcription value (Student's t test, P ≤ 0.05).

To study possible effects of cryptochrome-mediated light signals on the expression profiles of tomato photoreceptor genes, we compared mRNA levels in LD conditions in wt, in a *cry1a*- mutant [42] and in a *CRY2* transgenic over-expressor (*CRY2-OX*) [43]. The results indicated that loss of *CRY1a* as well as the over-expression of *CRY2* influenced the diurnal transcription profiles of several genes (Figure 6). In *cry1a-* or *CRY2-OX* plants, most tomato photoreceptor transcripts continued to cycle in LD conditions with the same phase as the wt, although with reduced or increased amplitude.

Major alterations involved *CRY1a*, *CRY2*, *PHYA*, *PHYB2* and *PHYF* transcripts. Interestingly, in the *cry1a-* background, the peak of the (non functional) *CRY1a* and of the *CRY2* transcripts was increased by about 2-fold with respect to the wt (Figure 6A). In *CRY2-OX* plants, as expected for the presence of the transgene, the *CRY2* mRNA was about 10–15 fold more abundant relative to the wt (Figure 6A). Remarkably, the *CRY2* transcript, over-expressed under the control of the *35S* promoter still showed vigorous LD cycles (Figure 6A). *PHYA* transcripts were altered in a similar way in both the *cry1a-* and *CRY2-OX* backgrounds, showing a decrease of mRNA abundance especially at ZT12 (Figure 6B). A different effect was observed for *PHYB2*, whose oscillation was slightly increased in *cry1a-* and reduced in *CRY2-OX* plants (Figure 6B). Finally, *PHYF* mRNA shows a dramatic increase in the *cry1a-* genotype (Figure 6B).

**Figure 7:**
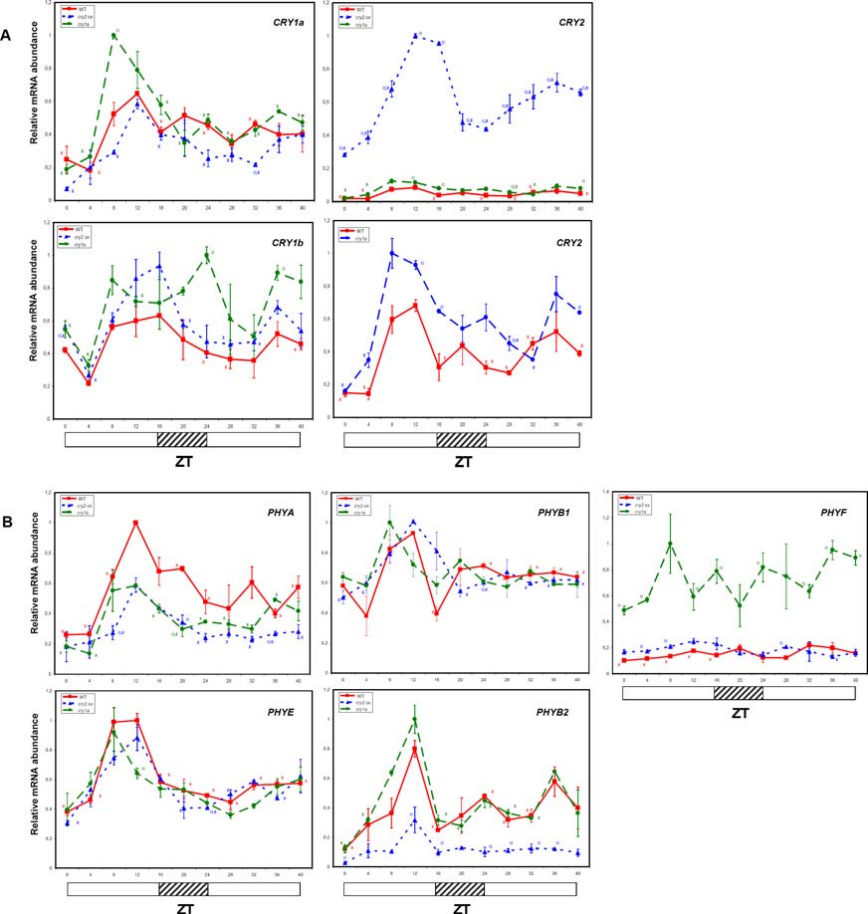
Effect of *CRY1a* loss-of function and *CRY2* over-expression on circadian expression of tomato cryptochrome (A) and phytochrome (B) genes in LL. Wt, *cry1a*- and *CRY2-OX* tomato plants were entrained under LD cycles and then transferred to LL. The abundance of the mRNAs were measured by QRT-PCR. Results are presented as a proportion of the highest value after normalization with β-actin. Open and hatched bars along the horizontal axis represent light and subjective night periods, respectively. Time points are measured in hours from dawn (zeitgeber Time [ZT]). An additional panel depicts *CRY2* transcript values in wt and *cry1a*- genotypes to avoid the masking effect of *CRY2-OX* values. Data shown are the average of two biological replicates, with error bars representing SEM. Circles (O) indicate time points of *CRY2-OX* and *cry1a-* genotypes, significantly different from the corresponding ones in wt genotype (Student's t test, P ≤ 0.05). For each genotype, X indicate time points significantly different from the highest transcription value (Student's t test, P ≤ 0.05).

In order to determine possible roles of cryptochromes on the function of the tomato circadian clock, we compared the changes in the mRNA abundance of photoreceptor genes, in wt, *cry1a-* and *CRY2-OX* plants grown under LL. Our results showed that loss of *CRY1a* as well as over-expression of *CRY2* influenced the circadian transcription profiles of a number of photoreceptor genes, including *CRY1a*, *CRY2*, *PHYA*, *PHYB2* and *PHYF* (Figure 7). As already observed in LD, the rhythm in *CRY2* expression was not affected by *CRY2* over-expression, although the transcript levels were 10–15 fold more abundant relative to those observed in wt (Figure 7A). Circadian oscillations of *PHYA* appeared to be repressed in *CRY2-OX*, following transfer to LL conditions, with a low amplitude oscillation remaining in *cry1a-* (Figure 7B). *CRY2* over-expression dramatically reduced the amplitude of *PHYB2* oscillations (Figure 7B). Finally, as for LD experiments, the *PHYF* transcription was dramatically increased under LL conditions in the *cry1a-* genotype (Figure 7B).

### Oscillation of tomato GI and LHC4 mRNAs and the effect of cryptochromes

**Figure 8:**
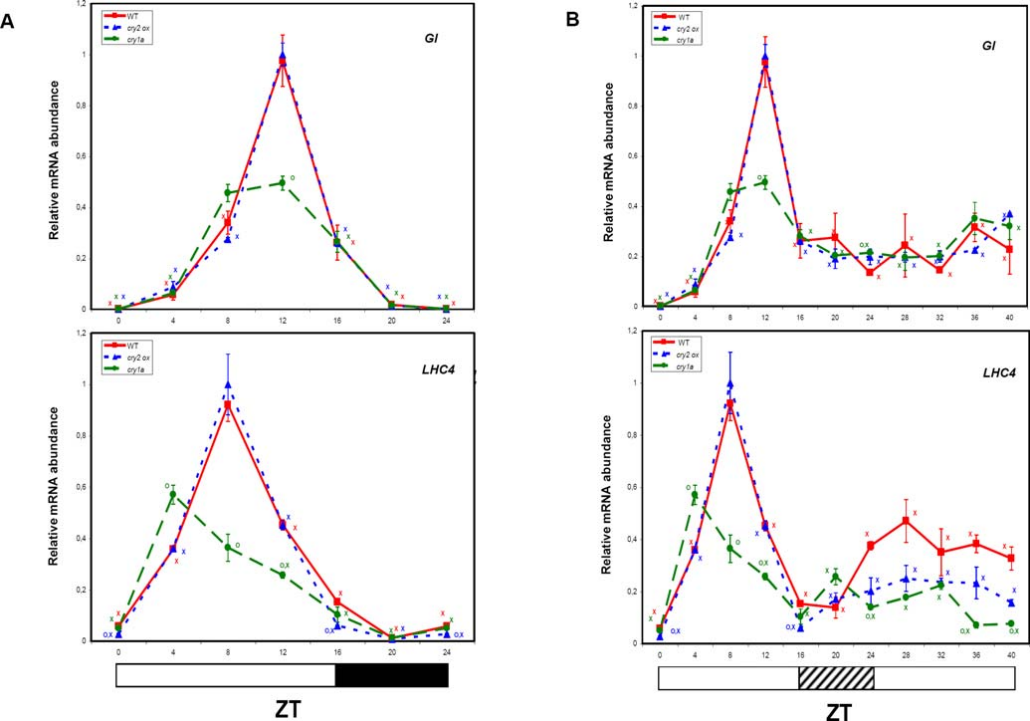
Effect of CRY1a loss and *CRY2* over-expression on light induced transcription of tomato *GI* and *LHC4* genes. Wt, *cry1a*- and *CRY2-OX* tomato plants were grown under LD (A) and LL (B) conditions. The abundance of the mRNAs of *GI* and *LHC4* genes were measured by QRT-PCR. Results are presented as a proportion of the highest value after normalization with β-actin. Open, closed and hatched bars along the horizontal axis represent light, dark and subjective night periods, respectively. Time points are measured in hours from dawn (zeitgeber Time [ZT]). Data shown are the average of two biological replicates, with error bars representing SEM. Circles (O) indicate time points of *CRY2-OX* and *cry1a-* genotypes, significantly different from the corresponding ones in wt (Student's t test, P ≤ 0.05). For each genotype, X indicate time points significantly different from the highest transcription value (Student's t test, P ≤ 0.05).

In LD conditions, *GI* transcripts oscillated about 800-fold, with a peak at ZT12, and a trough between ZT20 and ZT0 (Figure 8A). The *LHC4* peak occurred 4 h earlier (ZT8) (Figure 8A), and the trough 12 h later (ZT20) than *GI* (Figure 8A), with an 84-fold difference in transcript abundance. Interestingly, *LHC4* transcripts increased slightly in darkness from ZT20 to ZT24, showing an anticipation of “light-on” that is typical of circadian-regulated, particularly of *LHC* genes (Figure 8A) [53].

Under LL, *GI* and *LHC4* mRNA levels continued to cycle, although with a much reduced amplitude (Figure 8B), showing that transcription of these genes is partially controlled by the endogenous circadian clock. LL conditions affected both the amplitude/phase of oscillations as well as the abundance of the mRNAs (Figure 8B).

In the *cry1a-* genotype, both *GI* and *LHC4* transcripts showed a reduction of the peak at ZT12. In addition, a slight phase shift was evident which anticipated the transcription peak at ZT4 (Figure 8A), while no major alterations were observed in the *CRY2-OX* genotype.

Under LL conditions, neither *CRY1a* loss nor *CRY2* over-expression have dramatic effect on *GI* and LHC4 expression (Figure 8B).

## Discussion

### Diurnal expression of the tomato genome

Using the TOM2 microarray, we identified 1016 diurnally regulated genes, corresponding to about 15% of the spots that passed quality controls. Though by using high cutoff (>3x) values we might have excluded a certain number of transcripts cycling with lower amplitude, it is evident that in tomato, like in *Arabidopsis*, diurnal rhythms in gene expression affect a large portion of the transcriptome [36]. The majority of dirunally regulated genes showed a peak at midday (ZT8), while the other transcription peaks appeared evenly distributed at the other time points, supporting the occurrence of highly coordinated and alternated metabolic processes (see supplementary Data S1).

Given the cyclic nature of many physiological processes driven by photo- and thermocycles [54], it is expected that the majority of transcripts involved in the biosynthesis of mitochondrial and cytosolic proteins peak in the middle of the light phase (ZT8) (Figure 1A). This can be attributed to the fact that the biosynthetic processes correlated to photosynthesis and energy metabolism are usually more active in light hours. Similarly, the fact that several transcripts coding for proteins involved in transport, transferase activity and in the transcription control machinery were also abundant at dusk (ZT16) and during the night (ZT20) (Figure 1B) indicates that, during the hours of darkness, synthesis of these proteins is still active.

Several transcripts with higher levels during daylight (ZT8 to ZT16), grouping in clusters 2 and 4 (Figure 2), encode for protein elements involved in photosynthesis and stress response elements. The latter, that include a number of transcription factors – MYB, WRKY, bHLH, salt tolerance proteins, peroxidases, oxygenases and others, could have a major role to adapt tomato plants to day conditions, such as excess of light and higher temperatures.

Conversely, several transcripts relatively more abundant in during the dark phase (ZT16 to ZT20), grouped in clusters 2 and 3 (Figure 2), are related to biochemical processes occurring in darkness. We found genes involved in nitrogen and sulfur assimilation, as well as key genes involved cell wall loosening, such as xyloglucan endotransglycosylase and expansin. Most of these genes are already known to be down-regulated during daylight in *Arabidopsis* [35]. Thus, it seems that these processes are diurnally regulated in both *Arabidopsis* and tomato.

Plants need protection from the damaging effects of reactive oxygen species generated by the excess of light; in green tissues, carotenoids prevent the chlorophyll-photosensitized formation of highly destructive singlet oxygen by quenching the chlorophyll triplet states, scavenging reactive oxygen species. Furthermore, they have an antenna function and transfer the energy of absorbed light at the singlet excited state level to the chlorophyll system for the execution of photosynthesis [55]. Structural genes of the pathway appear to be diurnally regulated, although with different phases (Figure 2): *ZEAXANTHIN EPOXIDASE* (*ZEP*) is found in cluster 1, *VIOLAXANTHIN DE-EPOXIDASE* (*VDE*) and *PHYTOENE SYNTHASE* (*PSY*) in cluster 2, *β-CAROTENE HYDROXYLASE* (*CHY*) in cluster 3, *DEOXYXYLULOSE 5-PHOSPHATE SYNTHASE* (*DXS*) in cluster 4. Although light regulation of carotenoid gene transcription is a well known phenomenon [56], diurnal rhythms in gene expression have been reported, to date, only for *ZEP* [57]. Our data indicate that these rhythms are widespread in transcripts encoding carotenoid biosynthesis enzymes. This observation remains to be interpreted, in combination with data on the diurnal abundance and activity of the corresponding enzymes.

A good example of a possible coordinated response of tomato plants to abiotic stresses is given by the cyclic transcript oscillations of the DREB1A and DREB2 transcription factors (Figure 2, clusters 1 and 5). In *Arabidopsis*, *DREB1A* gene and its two homologs are implicated in response to low-temperature stress, in a manner independent of ABA, and its transcripts peak during the presumptive day, whereas expression of the *DREB2A* gene and its single homolog was induced by dehydration [47], [58]. Thus, it is plausible that the observed increment of *DREB* transcripts at ZT8 in tomato plants under LD conditions provide appropriate defense against changing temperature and dehydration occurring during light hours. The expression pattern of *DREB1A* evidenced a consistent increment of its transcripts at dark (ZT20) (Figure 2, cluster 5), possibly due to the decrease of ambient temperature at the presumptive dusk (ZT16) (see Material and Methods).

Several tomato homologues of the genes involved in the circadian clock feedback-loop in *Arabidopsis* [59] were found to oscillate in a similar phase in tomato: the morning element *LHY* was up-regulated ad dawn (Table 1); while *PRR7*, thought to establish a negative loop with *CCA1/LHY*, was more expressed during daylight (ZT8-ZT16) and down-regulated at dawn (Figure 2, cluster 4 and Table 1). *ELF4* and *GI* (discussed more in detail below), which are putatively involved in feedback-loops with *CCA1/LHY* and *TOC1/LUX*, respectively [33], were accordingly more expressed around dusk (Figure 2, clusters 6 and 4). These results suggest that the basic molecular machinery of the circadian clock is conserved in higher plants. Furthermore, the fact that a number of other elements, like *FKF1* [11] and *SPA1* [50] related to the input/output signalling of the *Arabidopsis* circadian clock, but also involved in flowering and light transduction, showed similar transcript fluctuations in tomato (Figure 2, cluster 4 and Table 1) suggests that molecular interactions between the clock core and input/output pathways are also partially conserved. However, it must be considered that our dataset is largely incomplete and does not represent the actual complexity of transcript network interactions described in *Arabidopsis*.

### Temporal modulation of cryptochrome and phytochrome transcripts

Previous experiments in *Arabidopsis* have established a fundamental role of phytochromes and cryptochromes in providing light input to the plant circadian clock [15], [53]. In tomato, as for *Arabidopsis*, we observed a bi-directional regulatory crosstalk between the clock machinery and photoreceptors which allowed the latter to determine significant changes on the temporal transcription pattern of genes under the control of the first.

As seen in *Arabidopsis* [53], tomato *PHY* and *CRY* genes followed a diurnal rhythm and exhibited maximum expression in the light phase (Figure 4A-D). Tomato photoreceptor transcripts, except for *CRY1b*, appeared to be synchronized and peaked during the presumptive afternoon, (Figure 4A-D). By contrast, in *Arabidopsis* gene expression trends are different between photo-stable and photo-labile photoreceptors. Indeed, light-stable photoreceptors are highly expressed at the beginning (*PHYC*, *PHYD*, and *PHYE*) or in the first half (*PHYB* and *CRY1*) of the light phase, while photo-labile *PHYA* and *CRY2* reach their maximum transcript abundance close to the end of the light interval. Unfortunately, data on the photo-stability of tomato photoreceptors are not yet available. The massive accumulation, in late afternoon, of most of the tomato photoreceptor transcripts, including *CRY-DASH* [60], might reflect the different photoperiodic behaviour of the two species (long-day for *Arabidopsis*, day-neutral for cultivated tomato).

The temporal regulation of *CRY1b* expression, whose mRNA was the most abundant among the analyzed photoreceptors, did not show remarkable fluctuations during the day, and was quite different from that of the other *CRY* genes (Figure 4A). Despite its high sequence similarity with *CRY1a*, this gene is not yet functionally characterized. The similarities of the expression patterns of *PHYA*, *CRY1a* and *CRY2* genes in both LD and LL (Figure 4B and Figure 5B), namely high levels of expression in the second part of the day (ZT8-16) and very low transcript abundances during the night, could be potentially related to overlapping functions and/or cooperation in their physiological roles.

*PHYB1* was the most abundant among phytochrome transcripts, followed by *PHYB2*, *PHYE* and *PHYA*, while *PHYF* is by far the least expressed phytochrome transcript in tomato green tissues (Figure 4). In LD, the expression peak of all phytochrome genes was between ZT8-ZT12 with no major phase differences. The amplitude of the oscillations was quite modest, with the sole exception of *PHYB2* that showed a 7-fold difference between through and peak transcript levels (Figure 4). These data contrast with a previous report [61] which evidenced a phase shift of about 10 hours between diurnal transcription rhythms of *PHYB1* and *PHYB2*. However, it must be taken in account that the authors used a quite different experimental set-up, with tomato plants grown in greenhouse and without supplemental illumination. This specific timing of transcript accumulation suggests that photoreceptor-mediated input signalling to the clock machinery may be particularly synchronized in tomato.

The fact that under LL all tomato cryptochromes plus *PHYA*, *PHYB2* and *PHYF* seem to keep their oscillations following a period close to 24 hours, though with lower amplitude and minor changes in the phase of the peaks (Figure 5A-D), hints that a circadian clock regulates the expression of these photoreceptors, as seen in the *Arabidopsis* closest homologs, *PHYA*, *PHYD* and *PHYF* [53]. In contrast, *PHYB1* and *PHYE* lose their rhythmicity in LL (Figure 5A, C), while the most closely related *Arabidopsis* homologs, *PHYB* and *PHYE* continue to cycle in LL with a peak at the beginning or in the first one-half of the light phase [53]. The different regulation in the two plants could reflect the different functional organization of the photoreceptor gene families. Differently from *Arabidopsis*, tomato flowering is day-neutral.

In LL, early into the presumptive night (ZT20), an increment in the *CRY2*, *PHYB2* and *PHYF* transcript levels with respect to the correspondent LD point was evident (Figure 5). This difference may be explained through postulating direct activation by light. The actual transcript levels appear to be then partially restored to the “normal” light/dark oscillation; this is possibly caused by some feedback action mediated by the clock machinery. This hypothetical feedback action is consistent with the model proposed by Tòth and colleagues [53], in which the photoreceptors send the “light-on” signal to the clock core, and the core regulates their expression, forming a regulatory loop. This regulatory loop could serve to increase the perception of resetting light signals at the right times, and to neutralize signals from non-predictable environmental cues, which could cause an incorrect resetting of the circadian clock.

### Influence of cryptochromes on temporal transcription of photoreceptor genes

In LD conditions, alterations in cryptochrome gene expression caused a minor increase of cryptochrome transcripts. The peak expression of *CRY1a* was incremented in *cry1a-* plants (Figure 6A); this could be the effect of an auto-regulatory feedback mechanism mediated by CRY1a and repressing the transcription of its own gene.

The increment of *CRY2* transcripts in *CRY2*-*OX* transgenic tomato is expected. However, the *CRY2* transcript, under the control of the 35S promoter, continues to cycle in both LD and LL (Figure 6A and Figure 7A). This strongly suggests that at least part of the *CRY2* oscillations are post-transcriptional. A similar situation has been observed in a transgenic line over-expressing *GI* [62]. To our knowledge, this is the first time that posttranscriptional (diurnal and circadian) oscillations are described in *CRY2*.

**Figure 9:**
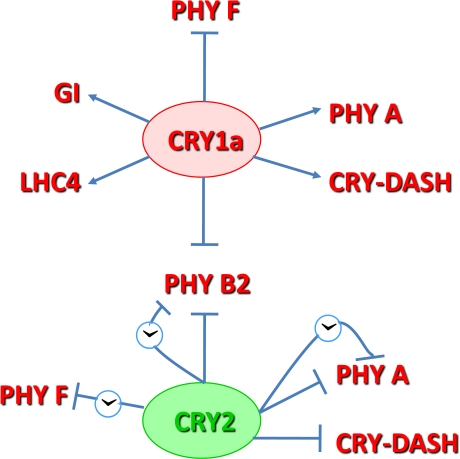
A model for genetic interactions among tomato CRY1a and CRY2 proteins and tomato photoreceptor, *LHC4* and *GI* genes, deduced from transcription experiments. The arrows represent a stimulatory effect, and the lines terminated with a bar represent an inhibitory effect. Positive and negative effects mediated by the circadian machinery are represented by the clock symbol.

Our data demonstrate that cryptochromes regulate phytochrome transcript levels, resulting in changes in transcript abundance, phase and cycling amplitude. Additionally, our data suggest that Cryptochromes 1 and 2 act cooperatively in repressing the transcription of *PHYA* and antagonistically on the transcription of *PHYB2*, which is promoted by CRY1a and repressed by CRY2 (Figure 6B and Figure 9). In *Arabidopsis*, there is evidence for a direct interaction between PHYA and CRY1, with PHYA mediating a light-dependent phosphorylation of CRY1 [14], and between PHYB and CRY2, with the CRY2 probably suppressing PHYB signaling [63]. Furthermore, in *Arabidopsis* CRY1 operates as a signal transduction component downstream of PHYA in light input to the clock [6]. In tomato, an additional level of suppression of PHYB signalling could be represented by the repressive action of CRY1a and CRY2 on *PHYB2* transcript levels (Figure 6B and Figure 9). Another interaction is the approximately 3–10 fold increase of *PHYF* transcripts at all time points in plants lacking a functional CRY1a.

Under LL conditions, transcriptional oscillations often became more perturbed and were sometimes difficult to interpret. However, the evident arrhythmicity of *PHYA* and *PHYB2* , but not *CRY1a* transcripts caused by the over-expression of CRY2 (Figure 7B) is quite intriguing and suggests that this condition specifically disrupts the output signal from the clock to *PHYA and PHYB2* (figure 9).

### Transcription rhythms of *LHC4* and *GI* are under cryptochrome control

In tomato wt plants, *GI* and *LHC4* transcripts cycled similarly to their putative counterparts in *Arabidopsis* [21], [64] with strong diurnal oscillations (about 800 and 90-fold, respectively). Lack of a functional CRY1a decreased the amplitude of the diurnal oscillation of both *GI* and *LHC4*, meaning that both genes, directly or indirectly, are activated by CRY1a (Figure 8A and Figure 9). A recent report showed that *Arabidopsis* mutant *cry1-cry2* plants displayed a severely reduced *GI* response to blue light, while CRY2 had no affect on the diurnal transcription of *GI* [65]. In agreement with these data, our results demonstrate that CRY1a plays a major role in the activation of tomato *GI* under high fluence white light. If we accept the hypothesis of GI as the “factor Y” in an interlocked feedback loop through light affecting TOC1 expression [33], we must conclude that CRY1a plays a major role in the input to the tomato circadian clock.

## Materials and Methods

Standard molecular biology protocols were followed as described in Sambrook and colleagues [66].

*Solanum lycopersicum* (cv *Moneymaker*), *cry1a-* and transgenic *CRY2-OX* [42], [43] were grown in a growth chamber for 28 days in LD conditions (16 h light-25°C/8 h dark-23°C). A light intensity of about 100 µmol m^−2^ s^−1^ was provided by Osram (Munich) 11–860 daylight lamps. For LL experiments, plants grown as described above for 28 days, were shifted to continuous light at the dawn of the 29th day. The aerial parts of three plants for each genotype (wt, *cry1a-* and *CRY2-OX*) were harvested at the times shown.

### Microarray analyses

Samples were assayed on the tomato TOM2 oligo-arrays printed at the University of Arizona which comprise contain 12,160 70-mer oligonucleotide elements (http://www.operon.com/arrays/oligosets_Tomato.php).

For each experiment, 2 µg of DNA-free total RNA was reverse-transcribed and amplified using the Aminoallyl Message Amp II kit (Ambion) following the manufacturer's instructions. 2 µg of amplified aminoallyl-modified RNA were labeled in the presence of Cy3 and Cy5 for 2 hours at room temperature. Unincorporated dyes were eliminated using RNeasy MinElute column (Qiagen) according to the manufacturer's specifications.

200 pmoles of purified Cy3- and Cy5-labelled aRNAs were combined in a buffer containing 2× SSC, 0.08% SDS and Liquid Blocking Reagent (GE Healtcare), and were dispensed over the microarray surface, and incubated at 55°C overnight with agitation. Slides were washed in decreasing SSC concentrations and 0.1% SDS at 55°C and room temperature, respectively. The last wash was carried out in 0.1× SSC at room temperature. The hybridization and post-hybridization washes were performed using an automatic hybridization station (HybArray 12, Perkin-Elmer). Hybridized microarrays were then scanned using ScanArray Lite (Perkin-Elmer) and the resulting Cy3 and Cy5 images were analyzed with the software ScanArray Express (Perkin-Elmer) in order to determine the Cy3/Cy5 spot intensities.

Raw hybridization signals were filtered by imposing a minimal signal/noise ratio of 2.0 and flagging the non-passed spots. In order to obtain a homogeneous dataset for all hybridized slides, we filtered microarray data imposing good quality spots to be present in at least three out of four hybridized slides (two dye-swap and two biological replicas, respectively) for each experimental point. Raw values were then normalized with the locally weighted linear regression (LOWESS) method using the 20% of data for smoothing [67] and gene expression analysis of the array data were performed using GeneSpring version 7.3 (Agilent).

After quality analysis and normalization (described above), we had a three-point LD time course with four microarrays per time point (2 independent biological replicates and 2 dye-swap experiments). For each of the transcripts which passed quality controls on the microarrays, a single factor ANOVA was performed across all three time points. Each time point was treated as a group, and arrays at each time point were treated as the individuals within that group. A nonadjusted ANOVA p-value of 0.05 or less was required for any particular transcript to pass the screen. After ANOVA-based statistical prescreening, genes showing equal to or more than 3-fold change in at least one of the three time points were considered diurnally regulated.

Moreover, in order to identify genes showing major transcript regulation at dawn (ZT0), for each of the transcripts which passed quality controls on the arrays, a Student's t test was performed across ZT8, ZT16, ZT20 together and their common reference ZT0. After above mentioned analysis, of the transcripts with a Student's t test p-value of 0.05 or less, that showing at least 3-fold change simultaneously at all time points were considered differentially regulated at dawn (Table 1).

Cluster Analyses were performed using Cluster and Treeview algorithms [68]. Microarray experiments have been deposited to the EBI public repository ArrayExpress (Accession number E-MEXP-1456).

### Quantitative RT-PCR

Total RNA (1 µg) was reverse-transcribed with oligo-dT and Superscript III (Invitrogen), according to the manufacturer's instructions. First strand cDNA (5 ng) was used as template for QRT-PCR. QRT-PCR assays were carried out with gene-specific primers, using an ABI PRISM 7900HT (Applied Biosystems) and the Platinum SYBR Green master mix (Invitrogen), according to manufacturer's instructions. PCR conditions were: 5 min at 95°C, followed by 45 cycles at 95°C for 15 sec, and at 58°C for 60 sec. At the end of the PCR, the thermocycler has been programmed to generate a thermal denaturation curve of the amplified DNA and to measure the melting temperature of the PCR product(s). The shape of the melting curve indicates whether the amplified products are homogeneous and the melting temperature provides confirmation that the correct product has been specifically amplified. Relative template abundance was quantified using the relative standard curve method described in the ABI PRISM 7900HT manual and the data were normalized for the quantity of the β-actin transcript [69]. A serial dilution of 10-, 100-, 1000-, 10000-, and 100000-fold of each studied gene fragment was used to determine the amplification efficiency of each target and housekeeping gene. At least three PCR runs were carried out for each cDNA to serve as technical replicates and two independent experiments were carried out by using two independent RNAs for each sample. Means from two independent experiments were subjected to SEM calculation, student's t test using PAST (http://folk.uio.no/ohammer/past/).

## Supporting Information

Data S1 (3.23 MB TXT)
